# Genome-Wide Identification and Analysis of the MADS-Box Transcription Factor Genes in Blueberry (*Vaccinium* spp.) and Their Expression Pattern during Fruit Ripening

**DOI:** 10.3390/plants12071424

**Published:** 2023-03-23

**Authors:** Xuxiang Wang, Qiaoyu Huang, Zhuli Shen, Ghislain Christel Baron, Xiaoyi Li, Xiaoying Lu, Yongqiang Li, Wenrong Chen, Lishan Xu, Jinchao Lv, Wenjian Li, Yu Zong, Weidong Guo

**Affiliations:** 1 College of Life Sciences, Zhejiang Normal University, Jinhua 321004, China; 2 Zhejiang Provincial Key Laboratory of Plant Biotechnology, Jinhua 321004, China; 3 Zhejiang Jinguo Environmental Protection Technology Company Limited, Jinhua 321000, China

**Keywords:** blueberry, genome-wide analysis, *MADS-box* genes, transcription factors

## Abstract

MADS-box is a class of transcriptional regulators that are ubiquitous in plants and plays important roles in the process of plant growth and development. Identification and analysis of blueberry *MADS-box* genes can lay a foundation for their function investigations. In the present study, 249 putative *MADS-box* genes were identified in the blueberry genome. Those *MADS-box* genes were distributed on 47 out of 48 chromosomes. The phylogenetic and evolutionary analyses showed that blueberry *MADS-box* genes were divided into 131 type I members and 118 type II members. The type I genes contained an average of 1.89 exons and the type II genes contained an average of 7.83 exons. Motif analysis identified 15 conserved motifs, of which 4 were related to the MADS domain and 3 were related to the K-box domain. A variety of cis-acting elements were found in the promoter region of the blueberry *MADS-box* gene, indicating that the *MADS-box* gene responded to various hormones and environmental alterations. A total of 243 collinear gene pairs were identified, most of which had a Ka/Ks value of less than 1. Nine genes belonging to *SEP*, *AP3*/*PI*, and *AGL6* subfamilies were screened based on transcriptomic data. The expression patterns of those nine genes were also verified using quantitative PCR, suggesting that *VcMADS6*, *VcMADS35*, *VcMADS44*, *VcMADS58*, *VcMADS125*, *VcMADS188*, and *VcMADS212* had potential functions in blueberry fruit ripening. The results of this study provide references for an in-depth understanding of the biological function of the blueberry *MADS-box* genes and the mechanism of blueberry fruit ripening.

## 1. Introduction

MADS-box is an important class of transcription regulators that is widely present in eukaryotes. The name of MADS-box originated from four proteins, *Saccharomyces cerevisiae* transcription factor (MCM1) [1], Arabidopsis flower homeotype gene (AGAMOUS) [2], snapdragon flower homeotype gene (DEFICIENS) [3], and human serum response factor (SRF) [4]. The amino acid sequences of those proteins contain a conserved region of about 60 amino acids, namely, the MADS-box domain [5]. *MADS-box* genes can be separated into type I and type II according to their sequence features [6]. Type I *MADS-box* genes in plants are also known as M-type genes and can be further divided into M_α_, M_β_, and M_γ_ subgroups [7,8]. Type II *MADS-box* genes mainly include a highly conserved MADS (M) region, moderately conserved keratin-like (K) region, relatively conserved intervening (I) region, and variable C-terminal (C) [8,9], which are also called MIKC-type. The MADS domain is involved in DNA binding and protein dimerization. The MADS domain and I domain jointly participate in the process of protein dimerization and affect the dimerization specificity. The K-box domain affects the interaction between MADS-box transcription factors. The C domain plays different functions in the various proteins, which enhance the function of the K-box domain or affect the DNA binding ability of the proteins. The MIKC-type *MADS-box* genes can be further divided into MIKC^c^ and MIKC* subgroups based on phylogenetic analysis [10]. Both type I and type II MADS-boxes contain a highly conserved MADS domain. The difference between these two types is that they contain divergent MADS domains. Most type II *MADS-box* genes include three other domains (‘I’, ‘K’, and ‘C’), while most type I *MADS-box* genes do not [11,12].

*MADS-box* genes play essential roles in plant growth, development regulation, and signal transduction. Previous studies about *MADS-box* genes mainly focused on the foundation of Arabidopsis female gametophytes [13], chloroplast formation [14], and embryo [15] and endosperm development [16]. The study interests of type I *MADS-box* genes were limited, possibly due to scarce transcription abundance and rarely reported functions [17]. By contrast, type II *MADS-box* genes attracted extensive and deep attention. Type II *MADS-box* genes were mostly recognized in the floral quartet model and the underlying ABCDE model of organ identity determination [18,19,20]. The expression of five classes of genes A, B, C, D, and E in plants separately or together contributed to floral organ development. The A function is mediated by *APETALA1* (*AP1*) and *APETALA2* (*AP2*), the B function by *APETALA3* (*AP3*) and *PISTILLATA* (*PI*), the C function by *AGAMOUS* (*AG*), the D function by *SHATTERPROOF* (*SHP*) and/or *SEEDSTICK* (*STK*), and the E function by *SEPALLATA* (*SEP*) [20]. Most of the five classes of genes belong to the *MADS-box* gene family. Type II *MADS-box* genes were also found to be involved in the growth and development of plant buds [21], stress resistance [13,22,23], and seed germination [24]. In addition, type II *MADS-box* genes were associated with fruit ripening [25]. The *SHATTERPROOF-like* gene (*FaSHP*) that belongs to the strawberry (*Fragaria* × *ananassa*) type II *MADS-box* was shown to regulate the ripening process of flesh fruit directly or indirectly through other transcription-factor-encoding genes [26]. The expression of *MADS-RIN* was required for tomato fruit ripening, which provides molecular insights into the developmental regulation of maturation [27].

To date, *MADS-box* gene families from numerous species were identified, including cabbage [28], tomato [29], sweet potato [30], willow [31], and wheat [32]. However, the blueberry (*Vaccinium* spp.) *MADS-box* gene family has not yet been analyzed. Blueberry is cultivated worldwide for its health benefits due to its abundant polyphenolic compound. Fruit ripening is the result of the joint regulation of multiple genes, which involves a series of physiological and biochemical changes. The ripening process of blueberry fruit is a crucial step for the formation and accumulation of fresh fruit flavor and other qualities. *MADS-box* genes were reported to be related to fruit development and ripening in a variety of plants, but their expression patterns during blueberry fruit ripening are still unclear. In this study, we performed genome-wide identification of the blueberry *MADS-box* genes. The chromosomal location, gene structure, phylogenetic relationships, and promoter cis-acting elements of *MADS-box* genes were analyzed. The biochemical features and conserved motifs of MADS-box proteins were also investigated. In addition, we screened *MADS-box* genes in accordance with the ripening process of blueberry fruit through transcriptomic data. The expression patterns of selected genes were validated using quantitative PCR. We aimed at providing references for understanding the potential function of blueberry *MADS-box* genes and the possible regulatory roles of *MADS-box* genes in blueberry fruit ripening.

## 2. Results

### 2.1. Identification of MADS-Box Genes in the Blueberry Genome

A total of 249 *MADS-box* genes (NCBI accession nos. OQ559686–OQ559934) were identified in the blueberry (*V. corymbosum* cv. ‘Draper’) genome. They were named from *VcMADS1* to *VcMADS249* according to the chromosome location and physical order on the chromosome (Supplementary Table S1). The amino acid number of MADS-box ranged from 135 (VcMADS98) to 672 (VcMADS195), with an average of 262 amino acids. The maximum and minimum molecular weight was observed at protein VcMADS195 (76.5 kDa) and VcMADS98 (15.5 kDa), respectively. The theoretical pI ranged from 4.56 (VcMADS228) to 10.4 (VcMADS208), with a mean value of 7.92. The instability indexes of MADS-box proteins were from 33.1 to 72.4. The results showed that 91.6% of MADS-box proteins were unstable with instability indexes larger than 40, while only 8.40% of MADS-box proteins had instability indexes less than 40. The predicted results of subcellular localization revealed that 64.7% of MADS-box proteins were localized in the nucleus. In addition, 18.5% and 3.21% of the MADS-box members were in chloroplasts and mitochondria, respectively.

### 2.2. Phylogenetic Analysis and Classification of MADS-Box Genes

**Figure 1:**
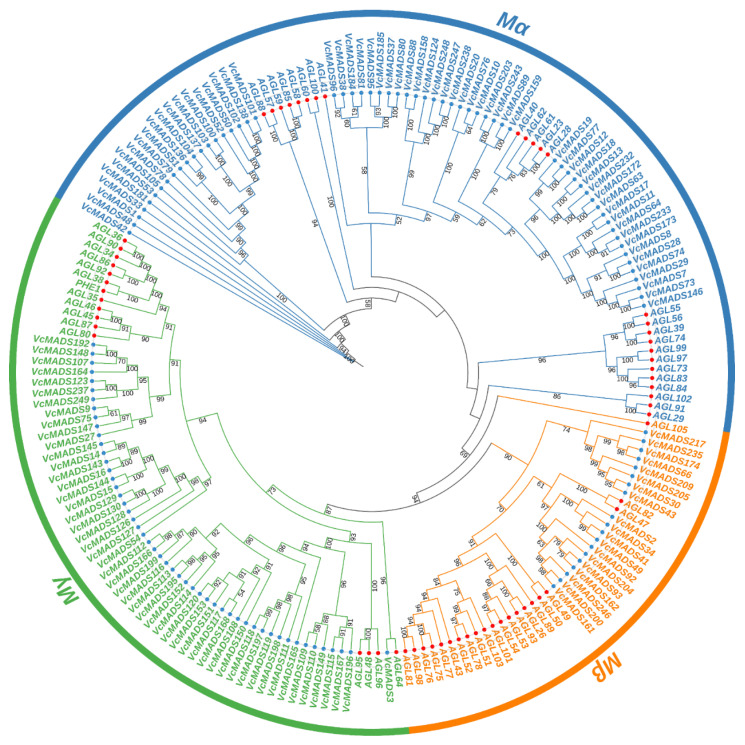
Phylogenetic tree of type I (M-type) MADS-box proteins from blueberry and Arabidopsis. MADS-box proteins of blueberry and Arabidopsis are marked with blue and red circles, respectively. The digits on the branches represent bootstrap values for 1000 replicates.

**Figure 2:**
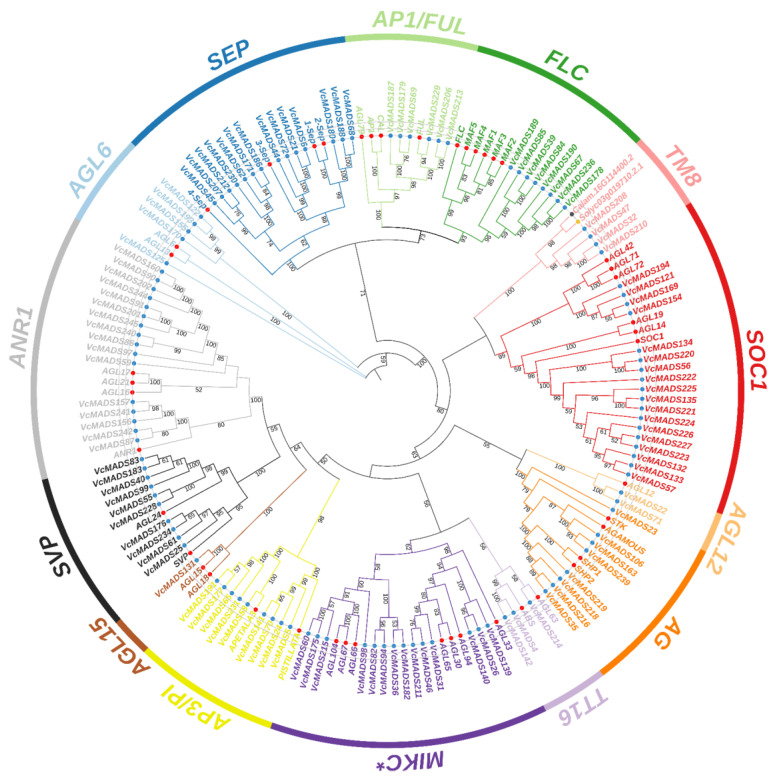
Phylogenetic tree of type II (MIKC-type) MADS-box proteins from blueberry, Arabidopsis, Calam.16G114400.2, and Solyc03g019710.2.1. MADS-box proteins of blueberry, Arabidopsis, Calam.16G114400.2, and Solyc03g019710.2.1 are marked by blue, red, brown, and orange circles, respectively. The digits on the branches represent bootstrap values for 1000 replicates.

To study the phylogenetic relationships of *MADS-box* genes between blueberry and Arabidopsis, preliminary evolutionary trees were built for 107 Arabidopsis MADS-box proteins and 249 blueberry MADS-box proteins. The results showed that 131 M-type (type I) and 118 MIKC type (type II) *MADS-box* genes were found in the blueberry genome. Different types of *MADS-box* genes in blueberry, together with Arabidopsis corresponding type I and type II *MADS-box* genes, Calam.16G114400.2, and Solyc03g019710.2.1, were taken to construct a phylogenetic tree. The results showed that the blueberry genome consisted of 61 M_α_ members, 19 M_β_ members, and 51 M_γ_ members (Figure 1). The blueberry MIKC-type MADS-box included 14 MIKC* and 104 MIKC^c^ members, among which MIKC^c^ could be further divided into 13 subgroups (Figure 2). Gene numbers in different subgroups ranged from 1 (*AGL15*) to 18 (*SOC1*), which indicated dramatic divergence among the subgroups. The subgroup *ANR1* contained 16 genes, followed by the subgroup *SEP* (14 genes). The subgroups *SVP* and *AP3*/*PI* consisted of 10 and 9 members, respectively. Subgroups *AG* and *FLC* both had eight genes, while the subgroups *AP1*/*FUL*, *AGL6*, *AGL12*, *TM8*, and *TT16* contained fewer members than the other subgroups, which just had six, five, two, four, and three genes, respectively.

### 2.3. Chromosomal Locations of MADS-Box Genes

**Figure 3:**
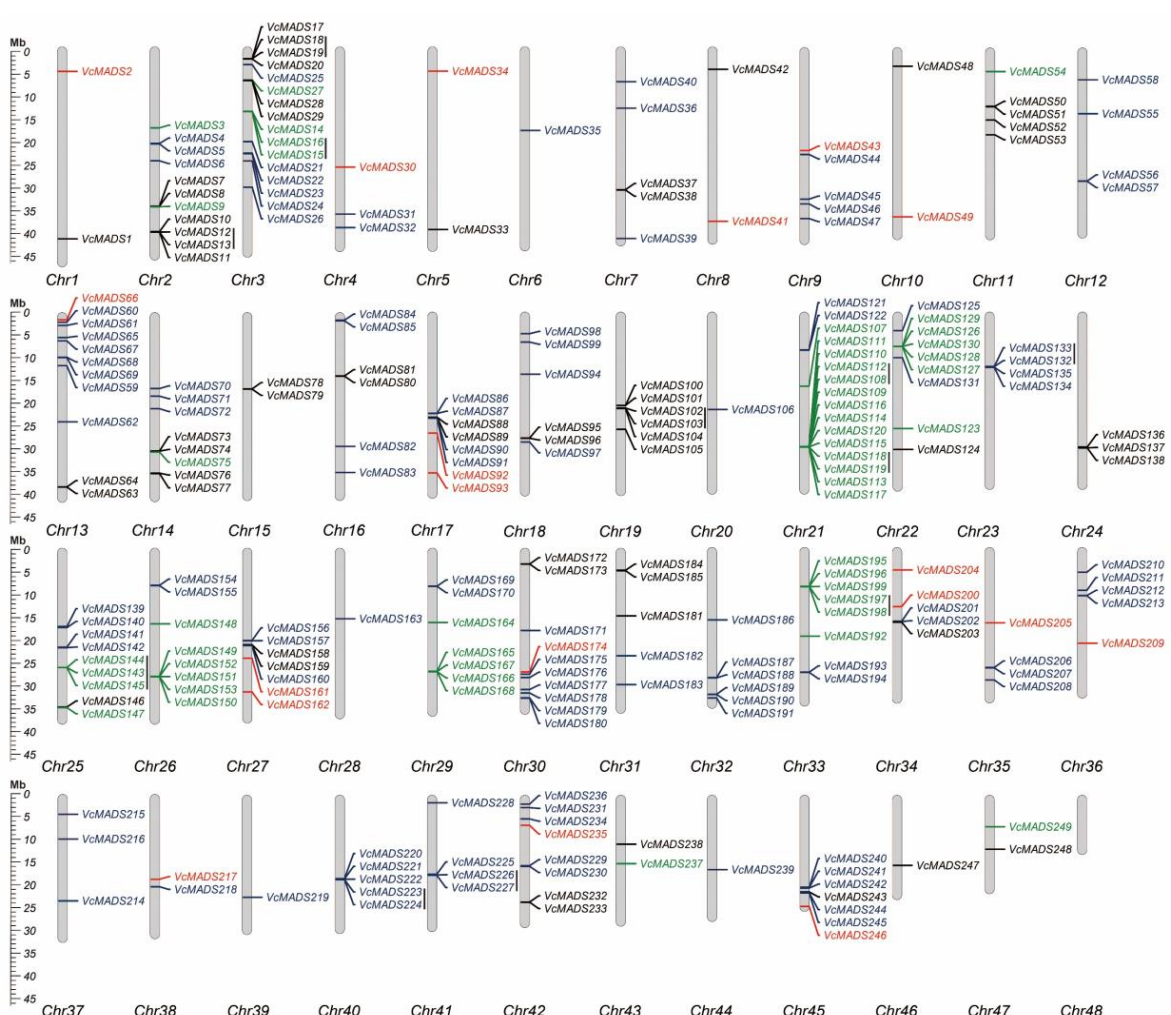
Chromosome location, distribution, and tandem duplication of *MADS-box* genes in 48 chromosomes of blueberry. M_α_, M_β_, M_γ_, and MIKC genes are shown in black, red, green, and purple, respectively. Vertical black lines indicate potential tandem repeat genes.

To gain insight into the chromosomal position of the 249 blueberry *MADS-box* genes, we analyzed their genomic distribution and found that they were unevenly distributed on nearly all chromosomes except chromosome 48 (Figure 3). High *MADS-box* gene abundance was found on chromosomes 2 (11 genes), 3 (16 genes), 13 (11 genes), 21 (16 genes), and 30 (10 genes), which contained more than 10 *MADS-box* genes. Whereas, only one *MADS-box* gene was discovered on each of chromosomes 6, 20, 28, 39, 44, and 46. Further analysis of chromosome location results showed that type I *MADS-box* genes only existed on eleven chromosomes. *MADS-box* members from the M_α_ subfamily were seen on chromosomes 15, 19, 24, and 46, which consisted of two, six, three, and one genes, respectively. Type II *MADS-box* genes were found on eleven chromosomes, i.e., chromosomes 6, 12, 20, 23, 28, 32, 39–41, 44, and 45. Furthermore, a total of 147 *MADS-box* genes formed 53 gene clusters distributed on 31 chromosomes, of which 50 gene clusters were composed of the same type of *MADS-box* genes. Thirty-four gene clusters contained *MADS-box* genes from the same subfamily, which indicated gene tendency and/or aggregate distribution tendency for blueberry *MADS-box* genes in the same subgroup.

### 2.4. Structure and Conserved Motif Analyses of MADS-Box Genes

**Figure 4:**
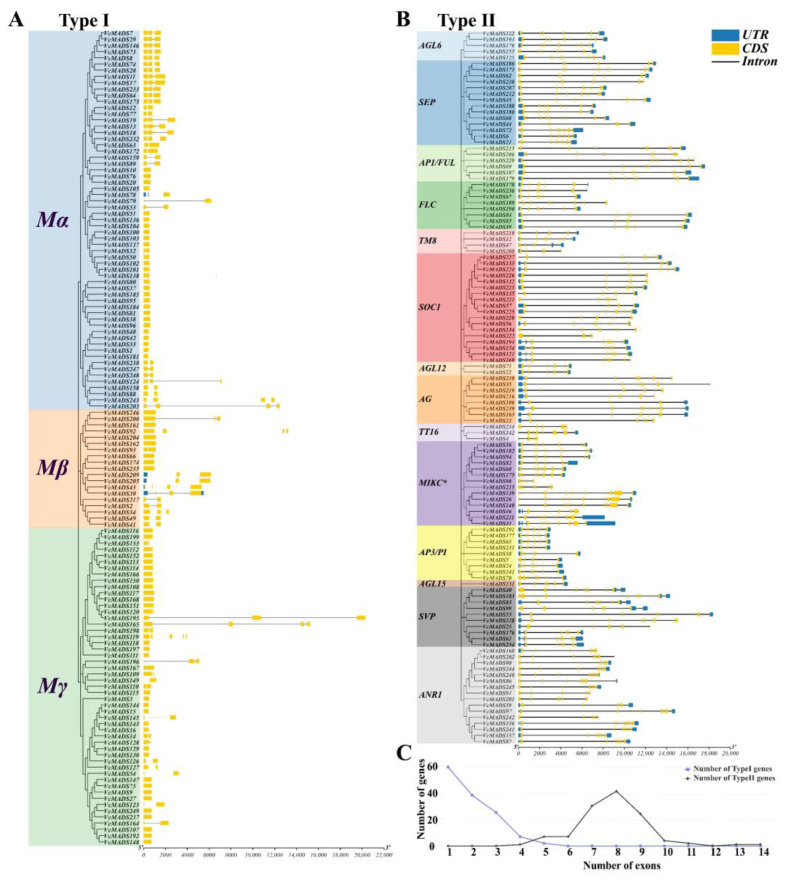
Gene structure analysis of 249 *VcMADS* genes. (**A**) type I *MADS-box* genes; (**B**) type II *MADS-box* genes; (**C**) exon number and their frequency of *MADS-box* genes, where blue and black lines indicate type I and type II *MADS-box* genes, respectively.

The length of the *MADS-box* genes was from 443 to 20,305 bp, with an average number of 5494 base pairs. Regarding the amino acid numbers, *VcMADS1* and *VcMADS195* were the shortest gene and longest gene, respectively. A significant difference was observed between the type I and type II *MADS-box* genes. In general, type I *MADS-box* genes were characterized by a shorter gene length and fewer exons (Figure 4A). The average length of the M_α_ subfamily genes was 1865 bp. *VcMADS203* (12,466 bp) and *VcMADS243* (11,980 bp) were significantly longer than the other genes. The M_α_ subfamily gene contained 1–4 exons, but all genes except *VcMADS13*, *VcMADS18*, *VcMADS203,* and *VcMADS243* had only 1–3 exons. It was found that there was a certain relationship between the gene length and the number of exons, and genes with a longer gene length tended to contain more exons, such as *VcMADS203* and *VcMADS243*. In addition, none of the M_α_ subfamily genes contained a UTR structure, except *VcMADS78*, which contained two five-prime UTR (untranslated region) structures. The M_β_ subfamily gene contained 1–4 exons, and the average length of the gene was 3182 bp, among which *VcMADS92* was 13,252 bp. Four genes (*VcMADS209*, *VcMADS205*, *VcMADS43*, and *VcMADS30*) contained UTR structures, and only *VcMADS30* contained a three-prime UTR. The M_γ_ subfamily genes contained 1–5 exons, but only *VcMADS119* and *VcMADS165* contained five exons. The average length of the M_γ_ subfamily genes was 1711 bp and the length of *VcMADS195* was 20,305 bp. In contrast to the M_α_ and M_β_ subfamilies, none of the M_γ_ genes contained UTR structures. The type II genes showed strong conservation within the same subfamily, and the genes in the same subfamily had a similar gene structure and length (Figure 4B). The MIKC* subfamily and AP3/PI subfamily were the two subfamilies with the highest and lowest average numbers of exons among the type II genes, respectively. The MIKC* subfamily genes contained 9.2 exons on average, and the AP3/PI subfamily genes contained 6.8 exons on average. Gene length analysis showed that the AP1/FUL subfamily (16,387 bp) and AP3/PI subfamily (3878 bp) were the subfamilies with the longest and shortest average gene length, respectively. Unlike type I genes, most type II genes contained UTR structures, and in addition to the ANR1 and MIKC* subfamilies, most of the genes belonging to other subfamilies contained both five-prime UTR and three-prime UTR structures. Most type I *MADS-box* (122, 93.1%) genes contained less than three exons and some genes (9, 6.90%) contained 4 or 5 exons, with an average of 1.89 exons (Figure 4C). In the type II *MADS-box* genes, the average exon number was 7.83. More than 95 (80.5%) genes contained 7–9 exons, while 15 (12.7%) genes had less than 6 exons and only 8 (6.78%) contained 10–14 exons.

**Figure 5:**
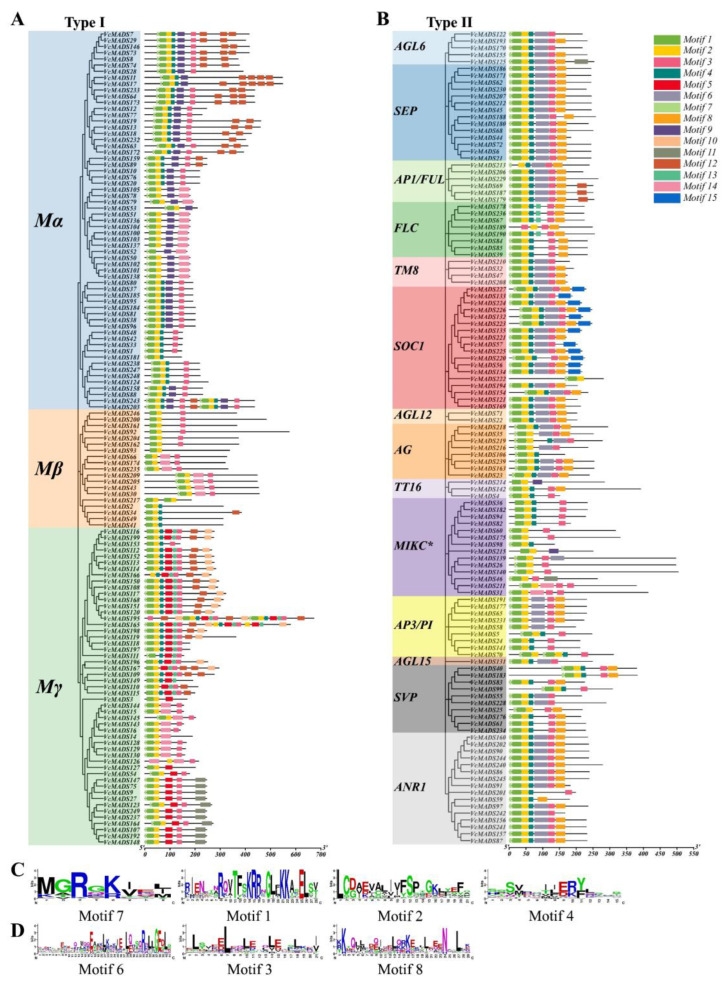
Conserved motif analysis of 249 VcMADS proteins. (**A**,**B**) Conserved motifs of type I and type II MADS-box proteins, respectively. (**C**) The 4 motifs located in the MADS domain region. (**D**) The 3 motifs found in the K-box domain region.

We performed a conserved motif analysis for VcMADS proteins with default parameter settings. Fifteen conserved motifs were uncovered in all VcMADS proteins (Table 1). In contrast to the type II genes, motifs 5, 9, and 10 were only found in the type I MADS-box proteins, where motif 9 was only found in the M_α_ subfamily and motifs 5 and 10 were only found in the M_γ_ subfamily. In addition, motifs 11, 12, 13, and 14 were mostly present in type I MADS-box proteins. Motif 11 was mainly distributed in the M_γ_ subfamily, motif 12 was mainly distributed in the M_α_ and M_γ_ subfamilies, and motif 14 was only present in the M_γ_ subfamily in type I MADS-box proteins (Figure 5A). Motifs 6, 8, and 15 were only found in MADS-box type II proteins, in which motifs 6 and 8 were widely distributed in type II proteins. However, FLC subfamily proteins lacked motif 6, MIKC* subfamily proteins lacked motif 8, and only one member contained motif 6. Motif 15 was found only in the SOC1 subfamily proteins (Figure 5B). Motifs 1, 2, 4, and 7 were in the MADS domain region. Motifs 3, 6, and 8 were found in the K-box domain region, of which motifs 6 and 8 were uniquely discovered in type II VcMADS proteins. All MADS-box proteins contained at least one of the four motifs, i.e., motifs 1, 2, 4, and 7, of which motif 2 was the most widely distributed one. Nearly all VcMADS proteins contained motif 2, with the exceptions of VcMADS52 and VcMADS111. Additionally, 90.4% of the proteins contained both motifs 1 and 2, and 69.1% of the proteins contained motifs 1, 2, 4, and 7.

We performed sequence alignment and generated sequence logos to check the motif conservation in the MADS and K-box domains. The motifs were ranked according to the position of the protein sequence (Figure 5C,D). The results show that motif 1 contained the most conservative amino acid sites (RQVTFSKRRNGLFKK), while motif 6 showed highly variable sites compared with the other motifs. Highly conservative leucine was found at many sites of motifs 2, 3, 4, and 8. Motif 7 comprised eight amino acids, which was the shortest one among the motifs. The first five amino acids (MGRGK) of motif 7 showed moderate conservation.

### 2.5. Cis-Acting Element Analysis of MADS-Box Genes

**Figure 6:**
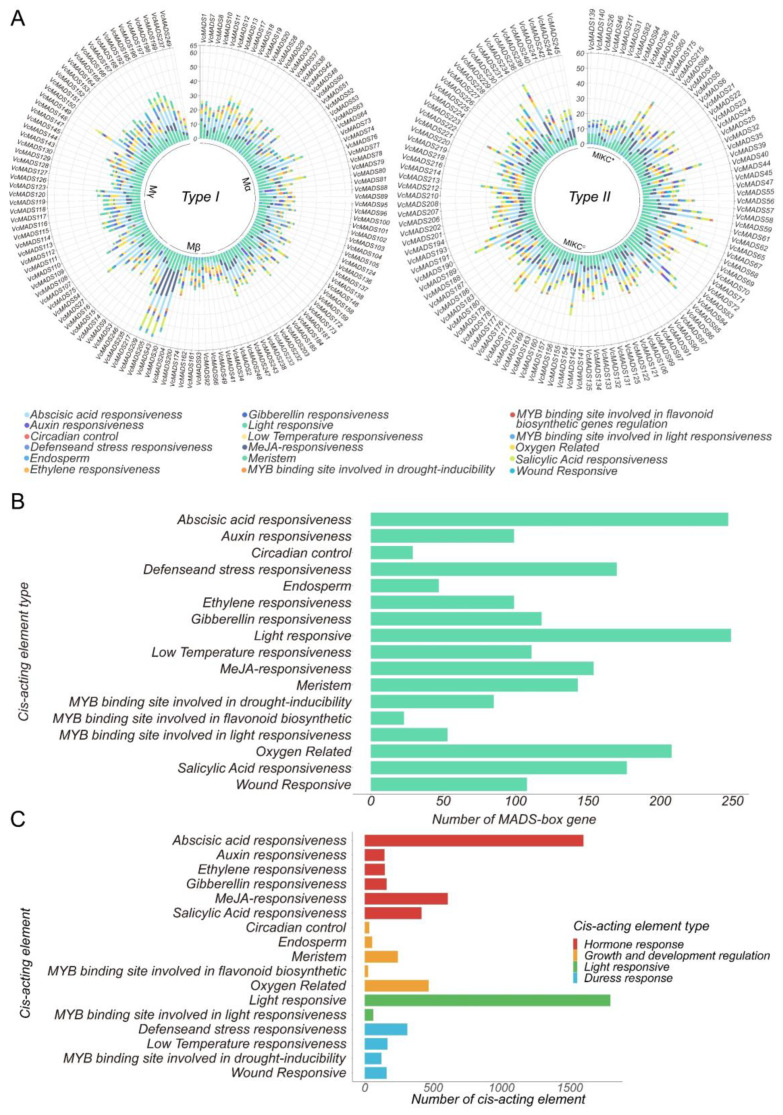
Cis-acting element prediction in blueberry *MADS-box* genes. (**A**) The number of cis-acting elements of type I and type II *MADS-box* genes. (**B**) The number of cis-acting elements distributed in *MADS-box* genes. (**C**) The total number of cis-acting elements, as well as the category.

Promoter cis-acting element analysis of *MADS-box* genes in blueberry showed that 6511 cis-acting elements were identified in the promoter regions of 249 *VcMADS* genes. We visualized the type and number of cis-acting elements on each gene and clustered them according to gene types and subfamilies (Figure 6A). The results showed that the average number of hormone (gibberellin, methyl jasmonate, and salicylic acid)-responsive elements of type II genes were higher than that of type I genes. However, the average number of auxin-responsive elements and ethylene-responsive elements was lower than that of type I genes. The resistance-related (MYB binding site, oxygen, and wound) response elements of the type II gene were also higher than that of type I genes. In addition, we further investigated the differences in the number of element types in different subfamilies. In type I genes, the average numbers of methyl jasmonate (MeJa)-responsive elements, MYB binding site elements, salicylic acid (SA)-responsive elements, low-temperature-responsive elements, ethylene (ETH)-responsive elements, and meristem regulatory elements in each M_β_ subfamily gene was higher than those in the M_α_ and M_γ_ subfamilies. Except for oxygen-related elements and ETH-responsive elements, there was no significant difference in the distribution of M_α_ and M_γ_ subfamily genes between element types (Figure 6B). We classified and counted the cis-acting elements in the promoter regions. The distribution of elements in the *VcMADS* genes was analyzed (Figure 6C). The elements can be divided into four categories, namely, light response, hormone response, stress response, and growth and development regulation. Hormone response cis-acting elements was the largest group within them, accounting for 47.2% of the total cis-acting elements. At least one kind of hormone response element was predicted in each gene. The number of cis-acting elements responding to abscisic acid (ABA), SA, andMeJa was higher than other responsive elements. A total of 1797 (27.6%) cis-acting elements associated with light response were found, including light response elements and MYB binding sites related to light response. Furthermore, the elements associated with light response were identified in all *VcMADS* genes. Stress response elements contained 758 (11.6%) components, which encompassed four subcategories: defense and stress response, low-temperature response, MYB binding site related to drought response, and wound response. There were 821 (12.6%) cis-acting elements that respond to the control of growth and development, including oxygen-related elements, circadian control, the endosperm, the meristem, and the MYB binding site involved in flavonoid biosynthetic genes regulation.

### 2.6. Collinearity and Evolutionary Analyses of MADS-Box Genes

**Figure 7:**
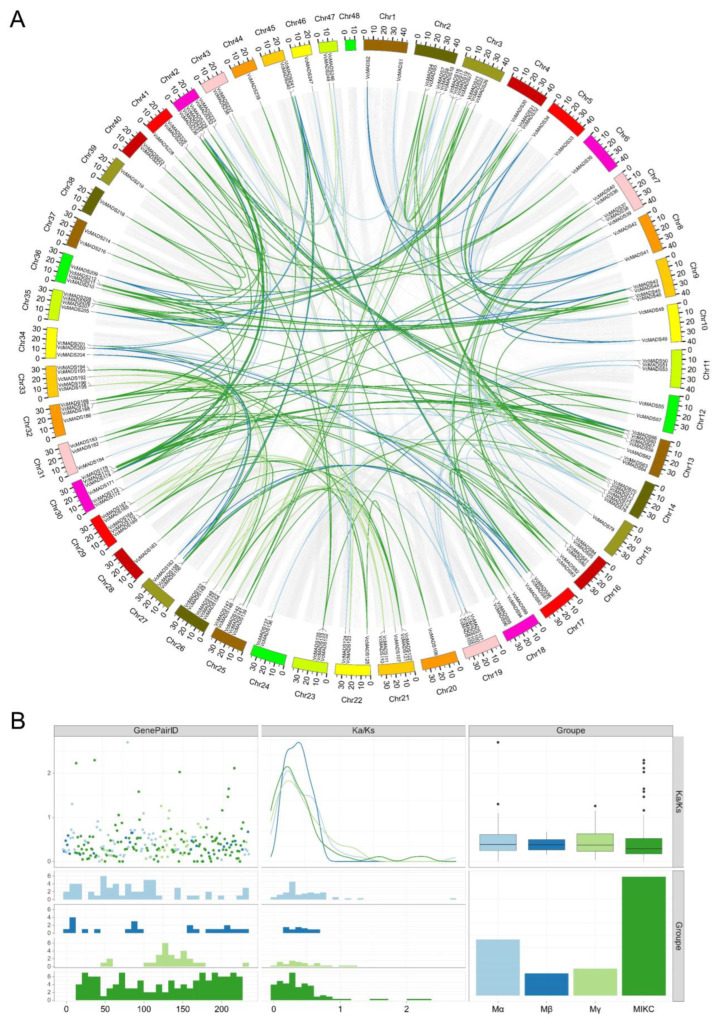
Collinearity of blueberry *MADS-box* genes and evolutionary selection pressure analyses. (**A**) The collinearity of the *VcMADS* genes. Light blue, dark blue, apricot, and green represent M_α_, M_β_, M_γ,_ and MIKC family gene pairs, respectively. (**B**) The Ka/Ks distribution of gene pairs. Gene pairs belonging to different subfamilies are shown in light blue, dark blue, apricot, and green, respectively.

The collinearity results showed that a total of 243 collinear gene pairs with whole-genome replication or segmental replication events were identified, among which 241 duplicate events occurred between different chromosomes and 2 duplicate events occurred on the same chromosome (Figure 7A). The results also indicated that 46.1% of the duplication events were found in type I *MADS-box* genes, involving 104 type I genes. A total of 103 type II *MADS-box* genes were discovered that experienced duplication events, accounting for 95.4% of all type II genes. Two or more genes that were located within a 200 kb region on the same chromosome containing were defined as tandem duplication. We found tandem duplication events for 12 VcMADS gene pairs (Figure 3), with 7 duplication events occurring in type I *MADS-box* genes and 5 duplication events occurring in type II genes. The Ka/Ks values of collinear gene pairs were calculated to unveil the evolutionary selection pressures of VcMADS genes (Figure 7B). The results indicated that most of the collinear VcMADS gene pairs had Ka/Ks values of less than 1, suggesting that most genes may have undergone selective pressure for purification during evolution.

### 2.7. Expression Patterns of MADS-Box Genes during Blueberry Fruit Ripening

**Figure 8:**
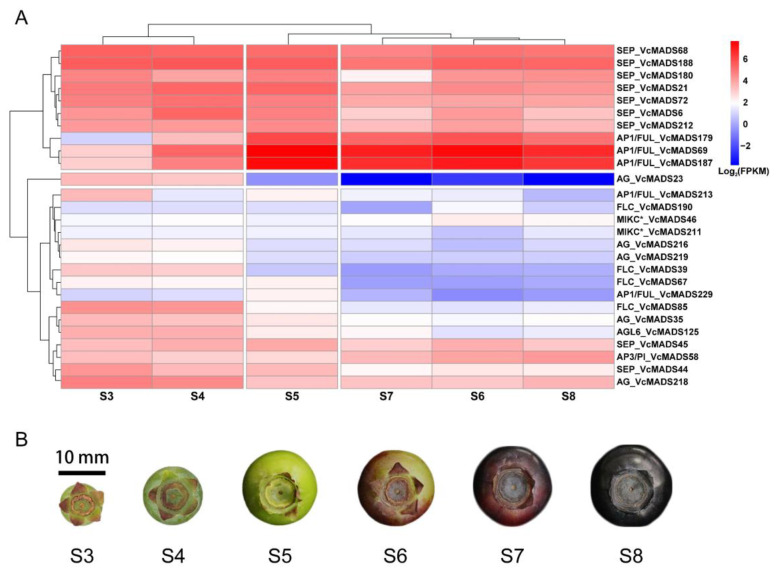
The heat map of 27 *VcMADS* genes highly expressed during blueberry fruit ripening. (**A**) Expression values were quantified as fragments per kilobase per million reads (FPKM) and normalized by taking logarithms. The rows and columns of the heat map were clustered. The row clustering clustered genes with similar expression patterns from S3 to S8, and the column clustering clustered similar stages based on the expression levels of 27 genes in different stages. (**B**) Six different stages of blueberry during fruit ripening. Blueberry fruit mainly expanded in the S3–S5 stages and changed color in the S6–S8 stages.

Based on the transcriptomic data of blueberry fruits, we found that 137 *VcMADS* genes showed non-zero FPKM values during six fruit developmental stages. We filtered 27 genes based on an FPKM threshold of 10 from stages S3 to S8 and a heatmap was plotted to show their expression patterns (Figure 8A). Most genes showed lower transcript abundances in stages S3 and/or S4 than those of stages S5, S6, and S8. Generally, genes from the same subfamily displayed similar expression patterns. For instance, *AG* genes were primarily expressed in the S3 and S4 stages, and *FLC* genes were commonly expressed from stages S3 to S5. The expression levels of type I *MADS-box* genes and type II genes differed greatly at the fruit maturation stages. Ten *VcMADS* genes were highly expressed during fruit ripening, of which seven genes belonged to the *SEP* subfamily and three genes belonged to the *AP1*/*FUL* subfamily. *SEP* genes and *AP1*/*FUL* genes exhibited distinct expression patterns at six stages of fruit ripening. *SEP* genes showed high expression levels and a general trend consistent with the fruit ripening process. The FPKM values of *AP1*/*FUL* subfamily genes increased sharply from stages S5 to S8 and reached summits in stage S5.

**Figure 9:**
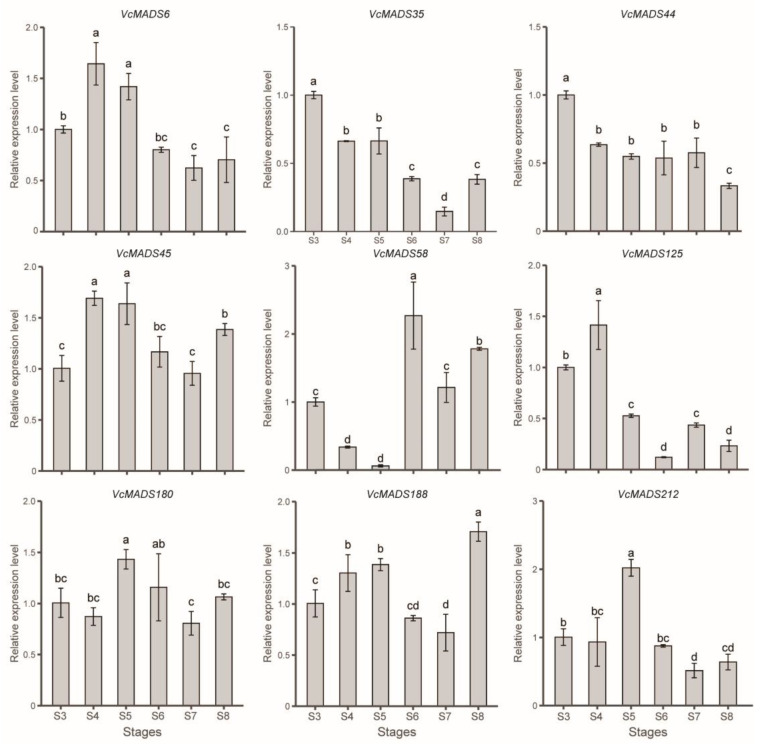
Relative expression of blueberry *MADS-box* genes in the fruit developmental stages. Lowercase letters represent significant differences. Different labeled letters for stages indicate significant differences in expression levels (*p* < 0.05, LSD test).

Nine genes—namely, *VcMADS6*, *VcMADS44*, *VcMADS45*, *VcMADS180*, *VcMADS188*, and *VcMADS212* of the *SEP* subfamily; *VcMADS35* of the *AG* subfamily; *VcMADS58* of the *AP3*/*PI* subfamily; and *VcMADS125* of the *AGL6* subfamily—were selected to check relative expression levels using quantitative PCR (qPCR). The expression patterns of those genes at different stages were consistent with those of the transcriptomic data (Figure 9). The results show that six *SEP* family genes (*VcMADS6*, *VcMADS44*, *VcMADS45*, *VcMADS180*, *VcMADS188*, and *VcMADS212*) were highly expressed during fruit ripening. Among them, the expressions of VcMADS6 and *VcMADS45* were higher in the S4 and S5 stages. *VcMADS44* had a high expression level in the S3 stage and a low expression level in the S8 stage. The expression level of *VcMADS180* did not show dramatic changes except during stage S5. *VcMADS188* was upregulated from stages S3 to S5, while it was downregulated in stages S6 and S7. The highest expression level of *VcMADS188* was observed at full ripening (S8 stage). The expression level of *VcMADS212* at stage S5 was significantly higher than that of other stages. *VcMADS58* belongs to the *AP3*/*PI* family and is mainly expressed in the S6–S8 stages and the highest expression level was in the S6 stage, with the relative expression reaching 2.27. Compared with other stages, the expression level of *VcMADS58* shows a very different expression level alteration. It decreased from stages S3 to S5 and sharply increased to its maximum in stage S6. *VcMADS35* belonged to the *AG* family and its expression level was downregulated from the S3 stage. The lowest expression level of *VcMADS35* was seen at full maturity. *VcMADS125* belonged to the *AGL6* family and was mainly expressed in the S4 stage, with low expression levels in stages S6 and S8.

## 3. Discussion

### 3.1. Gene Duplication Led to the Massive Replication of VcMADS Genes

*MADS-box* genes are ubiquitous in plants and play important roles in plant growth and development, stress response, signal transduction, and other processes. They were systematically identified and analyzed in some species, such as *Arabidopsis thaliana* (107 genes) [8], *Solanum lycopersicum* (131 genes) [29], *Zea mays* (211 genes) [33], and *Rhododendron ovatum* (77 genes) [34]. A total of 249 *VcMADS* genes were identified in the genome of blueberry, including 131 type I genes and 118 type II genes. It was found that the number of *MADS-box* genes in the blueberry was significantly higher than that of previously reported species. This is probably because blueberry has undergone multiple genome-wide doubling events during evolution [35]. Previous studies suggested that the blueberry experienced at least one paleohexaploidization event and two whole-genome duplication (WGD) events during speciation [34,36]. The most recent WGD event occurred about 9.04 million years ago as an independent doubling event [37]. Blueberry and *Rhododendron ovatum* shared the first two doubling events, but the number of *MADS-box* genes in *Rhododendron ovatum* was less than that in blueberry, suggesting that independent doubling events in blueberry evolution led to the massive expansion of *VcMADS* genes. Furthermore, the collinearity analysis identified 207 *VcMADS* genes with genome-wide or segmentary replication events, and 24 *VcMADS* genes with tandem replication events, indicating that small-scale segmentary and tandem replication events also contributed to *VcMADS* gene expansion in the blueberry genome. Tandem duplication events and segmental duplication events were both significant in the replication of the *VcMADS* gene, with segmental duplication events possibly acting as the primary driving force.

### 3.2. Retention and Potential Roles of TM8 Genes in Blueberry

Type II *VcMADS* genes were clustered into 14 subfamilies, including the *TM8* gene subfamily. *TM8* genes were absent in a variety of plants, including Arabidopsis and rice [38,39]. Although *TM8* genes were present in tomatoes and cucumbers, only some members were found. This is probably because *TM8* genes underwent multiple independent losses during the evolution of angiosperms and were not retained when coupling with genome duplication events [39,40]. We found four genes classified into the *TM8* subfamily in the blueberry genome, i.e., *VcMADS32*, *VcMADS47*, *VcMADS208*, and *VcMADS210*. As the ancient subfamily of the *MADS-box* gene family, few studies on the function of *TM8* genes have been conducted, with the exceptions of those involved in the development of female flowers and seeds [38,39]. Four *TM8* genes were expressed from stages S5 to S8 during berry ripening, and all reached the maximum during stage S6. This indicated that the *TM8* genes acted in pivotal roles in regulating fruit ripening. However, further studies should be conducted to confirm the regulation function of *TM8* genes.

### 3.3. The Possible Mechanism of Extranuclear VcMADS Protein Involved in Gene Regulation

Transcription factors are proteins that bind to specific genes and regulate their transcription, which usually occurs in the eukaryotic nucleus. In this study, 161 VcMADS proteins were predicted to be in the nucleus. There were 86 proteins found outside the nucleus, with 54 of them found in chloroplasts and mitochondria. Two proteins (VcMADS145 and VcMADS160) were predicted to be located at both the nucleus and cytoplasm. Chloroplasts and mitochondria contain DNA and RNA, and both include the machinery necessary for gene transcription. As a result, some *VcMADS* genes acted in roles in regulating photosynthesis and respiration-related genes. In addition, some transcription factor proteins can shuttle between the nucleus and cytoplasm through the phosphorylation/dephosphorylation nucleocytoplasmic trafficking mechanism [41]. It was reported that brassinosteroid (BR) treatment could recruit the cytoplasmic transcription factor BRASSINAZOLE RESISTANT1 (BZR1, a class of plant-specific transcription factors with noncanonical bHLH domains) into the nucleus and regulate the transcription process of BR response genes [42]. This indicated that the *VcMADS* proteins located outside the nucleus possibly enter the nucleus when needed for gene regulation. Nevertheless, experimental validation is necessary for testing this hypothesis in future studies.

### 3.4. Deletion of K-Box Domains in the MIKC* and the FLC Subfamilies

The type II (MIKC) *MADS-box* proteins generally contain four featured domains, namely, the MADS domain, I domain, K-box domain, and C domain. The MADS domain is the most conserved region in MADS-box proteins, and the K-box domain is moderately conserved in MIKC-type proteins. However, the function of the C domain has not been clearly defined due to its high variability [43]. Therefore, MIKC-type proteins are commonly considered to contain both MADS and K-box domains. The K-box was absent from 18 proteins among the 118 MIKC-type proteins identified in the present study. Four *FLC* subfamily members and all MIKC* subfamily members did not contain K-box domains. The absence of K-box domains in the MIKC* subfamily was reported in some species, including foxtail millet [23], American beautyberry [44], and litchi [45], which possibly related to MIKC* being a class of genes combining both features of type I (M_δ_) and type II genes. Some studies indicated that *MIKC^c^* genes may be the most ancient members of *MADS-box* genes, and type I genes probably evolved from *MIKC^c^* genes [10]. This suggested that MIKC* genes may be a class of transition genes retained after the loss of the K-box domain during the evolution of *MIKC^c^* genes. *FLC* genes were thought to be the most frequently lost *MADS-box* genes in plants [44], which indicated that the missing *FLC* genes contributed to K-box domain loss in the blueberry genome. The K-box domain contained K1, K2, and K3 subdomains, which corresponded to motifs 6, 3, and 8, respectively. Type II proteins lacking the K-box domain all contained one or two motifs, which is consistent with the idea that the K-box domain was lost during evolution. The contribution of the three subdomains to the K-box domain function was not the same. Some MADS-box proteins gained their K-box domain functions mainly through motifs within them [11]. This indicates that MIKC-type *VcMADS* proteins with a partial K-box domain possibly functionalize through conserved domains.

### 3.5. Potential Roles of MADS-Box Genes in Fruit Ripening in Blueberry

As an important transcriptional regulator in eukaryotes, MADS-box was widely studied in the process of fruit development. Two *FUL* genes in tomato, namely, *TDR4*/*FUL1* and *MBP7*/*FUL2*, were involved in cell wall modification, cuticle development, and volatile accumulation during fruit ripening [33]. The expression of four *SEP* genes in bananas caused an increase in ethylene content and affected banana maturation [46]. The expression of *SEP4-like* genes in strawberry was necessary for berry ripening [37]. The *MADS-box* gene is a critical component of the network that controls fruit ripening [40]. Blueberry fruit firmness changed dramatically during the fruit ripening stages. We found that the expression patterns of 16 genes potentially accompanied a decline in fruit firmness, among which 9 genes belonged to the *SEP* gene subfamily with high FPKM values. The results were further validated through quantitative PCR, which means that *SEP* genes may play an important role in the ripening process of fruits, which is consistent with the results of the previous study [47]. The three *AP1*/*FUL* genes were highly expressed from stages S5 to S8. This indicates that the three *AP1*/*FUL* genes were also significant for fruit ripening. However, the specific regulatory mechanisms of the *MADS-box* gene in the blueberry fruit softening process need to be explored further in the future.

## 4. Materials and Methods

### 4.1. Identification of MADS-Box Genes in Blueberry

The amino acid sequences of the blueberry cultivar ‘Draper’ was downloaded from the *Vaccinium* Genome Database (https://www.vaccinium.org/ (accessed on 5 September 2022) [35]. A local protein database was constructed using the MAKEBLASTDB function in the BLAST+ 2.13.0 toolkit. The MADS protein sequences (M-type_MADS and MIKC_MADS types) of Arabidopsis thaliana were downloaded from Plant Transcription Factor Database (http://planttfdb.gao-lab.org/ (accessed on 7 September 2022)) and used to query the blueberry MADS-box homologs. Hit targets with an e-value less than 1 × 10^−20^ were kept for further analyses. Other parameters of BLASTP were kept as default. In addition, we performed HMMER [48] analysis using seed alignment of the MADS-box domain (PF00319), which was obtained from the Pfam database (https://pfam.xfam.org/ (accessed on 8 September 2022)) [49]. Blueberry MADS-box gene candidates were screened by using an e-value threshold of 1 × 10^−10^. We merged the outputs from BLAST and HMMER and removed redundant results for downstream analyses. The conserved domains of candidates were verified using the online tool NCBI Conserved Domain Search (https://www.ncbi.nlm.nih.gov/Structure/cdd/wrpsb.cgi (accessed on 12 September 2022)). The members that contained a full MADS domain were recognized as blueberry *MADS-box* genes. The biochemical properties of blueberry MADS-box proteins were analyzed with ExPASy protparam (https://web.expasy.org/protparam/ (accessed on 14 September 2022). Subcellular localization of blueberry MADS-box proteins was predicted using WoLF PSORT (https://wolfpsort.hgc.jp/ (accessed on 15 September 2022).

### 4.2. Phylogenetic Analysis and Classification of Blueberry MADS-Box Genes

The amino acid sequences of Arabidopsis MADS-box were downloaded from TAIR (https://www.arabidopsis.org/ (accessed on 26 September 2022) and were aligned with the blueberry MADS-box proteins using ClustalW [50] with default parameters. Using the maximum likelihood method, a primary phylogenetic tree was constructed using IQ-TREE (multicore version 2.2.0) [51] with 1000 bootstraps. Based on the classification rules of the Arabidopsis MADS-box, the blueberry MADS-box was divided into type I and type II in the primary tree. Type I and II MADS-boxes in Arabidopsis were used to build the type I and type II phylogeny trees with corresponding blueberry MADS-boxes. In addition, *TM8* is a class of genes belonging to the type II MADS-box, but such genes were lost in a variety of plants and are not present in Arabidopsis. To determine the presence of the *TM8* gene in blueberry, we added two TM8 genes, namely, Solyc03G019710.2.1 in tomato [29] and Calam.16G1144000.2 in American beautyberry [44], as grouping criteria for *TM8* class genes when constructing the type II phylogenetic tree. The online tree plotting tool iTOL (https://itol.embl.de/upload.cgi (accessed on 29 September 2022) [52] was used to complement the phylogenetic tree.

### 4.3. Chromosomal Location and Conserved Motif Analyses of MADS-Box in Blueberry

The distribution information of the blueberry *MADS-box* gene on the chromosome was extracted from the genome general feature file. We used the online tool MG2C (http://mg2c.iask.in/mg2c_v2.1/ (accessed on 2 October 2022) to draw the chromosome location of blueberry *MADS-box* genes. MEME (http://meme-suite.org/ (accessed on 6 October 2022) [53] was used to analyze the conserved motif of blueberry *MADS-box* genes and the maximum number of conserved motifs was set to 15. The gene structures and conserved motifs of the MADS-box family in the blueberry were visualized using Tbtools [54].

### 4.4. Cis-Acting Element, Collinearity, and Evolutionary Selection Analysis of Blueberry MADS-Box Genes

We extracted 1500 bp sequences of blueberry *MADS-box* genes upstream of the start codon using Tbtools. Sequences were submitted to the website PlantCare (http://bioinformatics.psb.ugent.be/webtools/plantcare/html/ (accessed on 15 October 2022) [55] for cis-acting element analyses. MCScanX [43] and Circos [56] were used to estimate the collinearity relationships of *MADS-box* genes and to plot the collinearity figure, respectively. Furthermore, to understand the selection pressure on the *MADS-box* genes in blueberry, Ka/Ks values of MADS-box gene pairs were calculated using KaKs_Calculator [57].

### 4.5. Plant Materials

The southern highbush blueberry (*V. corymbosum* cv. ‘Star’) used in this study was collected from the blueberry Germplasm Resource Nursery of Zhejiang Normal University. According to Zifkin et al.’s method for identifying the developmental stages of blueberry fruits [58], fruit samples were collected from stages S3 to S8. The berries were immediately frozen in liquid nitrogen and stored at −80 °C for downstream studies. Total RNA from blueberry fruits was extracted using the CTAB method. Synthesis of the first strand of complementary DNA was performed using a reverse transcription kit (Vazyme, Nanjing, China).

### 4.6. Expression Pattern of MADS-Box Genes during Blueberry Fruit Ripening

Transcript abundance of blueberry fruits at different developmental stages, represented by fragments per kilobase per million reads (FPKM), was obtained from our previous transcriptomic data. *MADS-box* genes that showed significantly different expression levels between ripening stages were screened. Nine genes were selected for the qPCR assay to check their expression pattern. Primers were designed using NCBI Primer-BLAST and were synthesized by the Sangon Biotech Company (Shanghai, China). The specificity of primers was verified using agarose electrophoresis and dissolution curves. qPCR was performed using the QuantStudio™ 1 real-time quantitative system. The total volume of the reaction system was 10 μL with 5 μL 2× SYBR Green qPCR premix, 2 μL double distillation H_2_O, 1 μL complementary DNA, and 1 μL for forward and reverse primers. The *VcGAPDH* gene was chosen as the internal reference gene. The primer sequences used in the current study are shown in Table S2. The procedure for qPCR was 10 min at 95 °C, followed by 40 cycles of 95 °C for 30 s, 57 °C for 30 s, and 72 °C for 30 s. The 2^−ΔΔCt^ method was used to calculate the relative expression, with three biological replicates per sample. R scripts were used to analyze the relative expression and to draw the histogram.

## Figures and Tables

**Table 1:** Conserved motifs of blueberry MADS-box proteins.

Motif	Length	Amino Acid Sequence
1	28	RIENKTNRQVTFSKRRNGLFKKASELSV
2	21	LCDAEVALIVFSPTGKLYEFG
3	21	ELSVEELEQLEKQLETSLKEI
4	15	SSSVRGIIERYLSMS
5	29	MLTHEGYLTQRIASEIKRNDKAKKKNDMK
6	41	QQDHTLEEKEAQFWQQEAAKLKAKJEALZRSQRNLLGEDLG
7	8	MGRGKIEL
8	29	KKTQLLKDZIQELQRKEKLLEZENKTLLK
9	29	NIVVAQRNENLRLLNMQLTNAQAELEAEK
10	29	YQQDIPCGPSWVISDLEIASPEHHVAGNM
11	41	MQSGPQWFTDWVNNPNENMGFGGDDQMMLPFGDGHSAMWSS
12	29	PPPPPPPPLEEREGGPHQQAAAPPPPPPP
13	15	QEDMNKIYEGSLDLG
14	41	AZQKIDEYRBYPVPEQKKRLLELEGFINNRLEKJEEKVTKK
15	37	QEPSEEIATVALTNSREDSDVETELFIGQPLGRIKPT

## Data Availability

The RNA-seq datasets presented in this study were deposited in the National Genomics Data Center (NGDC) database and are accessible through GRA accession code CRA010224 (https://bigd.big.ac.cn/gsa/browse/CRA010224, accessed on 19 March 2023).

## Supplementary Materials

The following supporting information can be downloaded from https://www.mdpi.com/article/10.3390/plants12071424/s1, Table S1. Protein information analysis of MADS box gene family members in blueberry. Table S2. Primers used in this study.
